# Bogus Diplomas

**Published:** 1873-02

**Authors:** 


*Bogus Diplomas.*—The *London Lancet* under the title of "Philadelphia Degrees," calls attention to the traffic in diplomas, which it appears is still going on, notwithstanding the action of the Pennsylvania Legislature in repealing the charters of certain disreputable institutions in the city of Philadelphia. Why the buyers of such frauds cannot themselves prepare equally valuable parchments, and thereby save all but the cost of material, is a mystery. For the notoriety of the traffic as well as the terms upon which these fraudulent diplomas are offered, puts all excuse as to ignorance on the part of the purchasers, out of the question:

"We have information, which seems to come from an authentic source, that owing to one most important portion of the Acts repealing the charters of certain American colleges that trafficked in degrees having been undesignedly omitted, advantage has been taken of the omission to continue the traffic in England. A 'Dr.' agent from New York has been calling on medical men and others, representing that diplomas can be received from the Pennsylvania University for ten dollars.

Circulars to a similar effect have been sent to us in great numbers. After recent exposure there is no excuse for any who take an American degree *in absentia* or on terms of purchase. We are informed that the defect in the Acts referred to will be speedily remedied. The sooner the better. We trust that the various States of the Union will take this whole subject into consideration, and pass such laws as will secure the respectability of degrees, which has been so rudely damaged."

